# Multi-Septate Gallbladder in a Patient With Acute Cholecystitis

**DOI:** 10.7759/cureus.46762

**Published:** 2023-10-09

**Authors:** Kasturi Desai, Sanjeev Gianchandani

**Affiliations:** 1 Medicine, Jawaharlal Nehru Medical College, Datta Meghe Institute of Higher Education and Research, Wardha, IND; 2 General Surgery, Jawaharlal Nehru Medical College, Datta Meghe Institute of Higher Education and Research, Wardha, IND

**Keywords:** biliary pain, lap cholecystectomy, mrcp imaging, severe acute cholecystitis, honeycomb gallbladder, multiseptate gallbladder

## Abstract

A very uncommon congenital defect called multi-septate gallbladder (MSG) typically results from wrinkling of the gallbladder membrane or insufficient vacuole formation of the growing bud of the gallbladder. The many septa giving MSG its honeycomb appearance cover the whole lumen of the gallbladder. MSG, hyperplastic cholecystitis, and cholecystitis are a few causes of this ultrasonography finding. A large number of patients describe having persistent stomach issues, including discomfort in the epigastrium, and frequent episodes of discomfort in the abdomen with episodes of nausea. The gallbladder's reduced motility is caused by septa, which causes a halt in the passage of bile and may be the cause of persistent stomach discomfort. The purpose of this report is to provide readers with a better knowledge of this ailment and its recommended course of treatment. We are describing a case of a MSG in a 22-year-old patient. We additionally included a few instances for evaluation and analysis. According to the research that is currently accessible, congenital MSG is most likely caused by the gallbladder wall being pushed into its cavity, creating septa that contain muscle fibers. Alternative imaging techniques like magnetic resonance cholangiopancreatography (MRCP) and endoscopic retrograde cholangiopancreatography (ERCP) are the best tools to make the diagnosis. The effectiveness of medical interventions is unknown; however, cholecystectomy has completely resolved symptoms in people.

## Introduction

A congenital abnormality known as the septate gallbladder involves the gallbladder having one or many septa that divide its cavity into distinct sections. Tiny openings inside the gallbladder may allow for interaction among its many components. In the majority of cases, multi-septate gallbladder (MSG) has been classified as a congenital abnormality, whereas in some instances, it has been classified as an acquired phenomenon. Most often non-symptomatic, the illness is only unintentionally picked up on scanning. The condition was also documented with several distinct symptoms and treatments. Although the illness may occur in newborns and elderly individuals, gender is not a risk factor. The most typical initial symptom is discomfort in the abdomen; however, it can often occur without any other complaints. Only imaging can be used to diagnose MSG, and ultrasound seems to be a reliable and practical method of diagnostics. Ultrasonography (USG) assessment of the gallbladder is typically enough. The most accurate and least intrusive way to identify a MSG is to combine USG and magnetic resonance cholangiopancreatography (MRCP) [1].

## Case presentation

A 22-year-old female patient was hospitalized in our department a few years ago complaining of persistent abdominal pain. She additionally complained of nausea and dyspepsia in addition to right hypochondria discomfort after fatty meals. The upper right quadrant of the body was sensitive, and the Murphy's sign was positive. No prior episodes of a high temperature, throwing up, diarrhea, or urinary manifestations were noted. Her vital signs were within normal ranges for her age on her initial clinic visit. Her torso was tender, lacking any pain, lumps, or organomegaly during the medical checkup. Laboratory analysis revealed a higher white blood cell count and CRP. There were no aberrant results from further biochemical tests such as the complete blood count, liver function test, alkaline phosphate, erythrocyte sedimentation rate, bilirubin, or transaminases. Neither fever nor jaundice was seen in the patient. No relevant illnesses were present in the family history. Blood problems and infectious infections were excluded.

**Figure 1:**
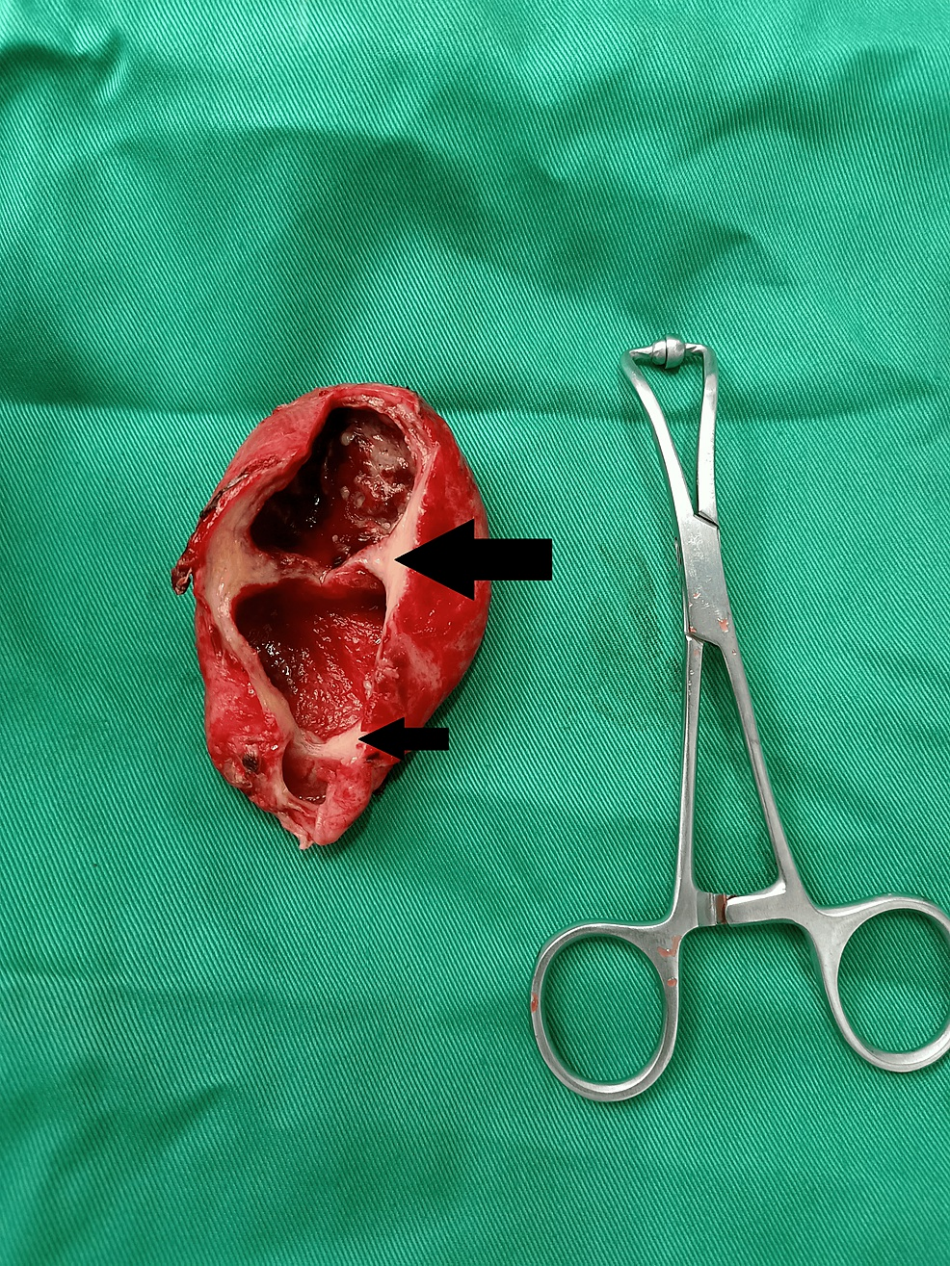
Multiple septations in the gallbladder A horizontal septum was visible upon close inspection of the specimen. The gallbladder was split into two sections by septa which are shown by arrows. The entire gallbladder wall had signs of inflammatory alterations. Due to a lack of resources, the surgeon utilized a 5-inch instrument to compare the gallbladder's size. The gallbladder measures approximately 3 inches in length.

**Figure 2:**
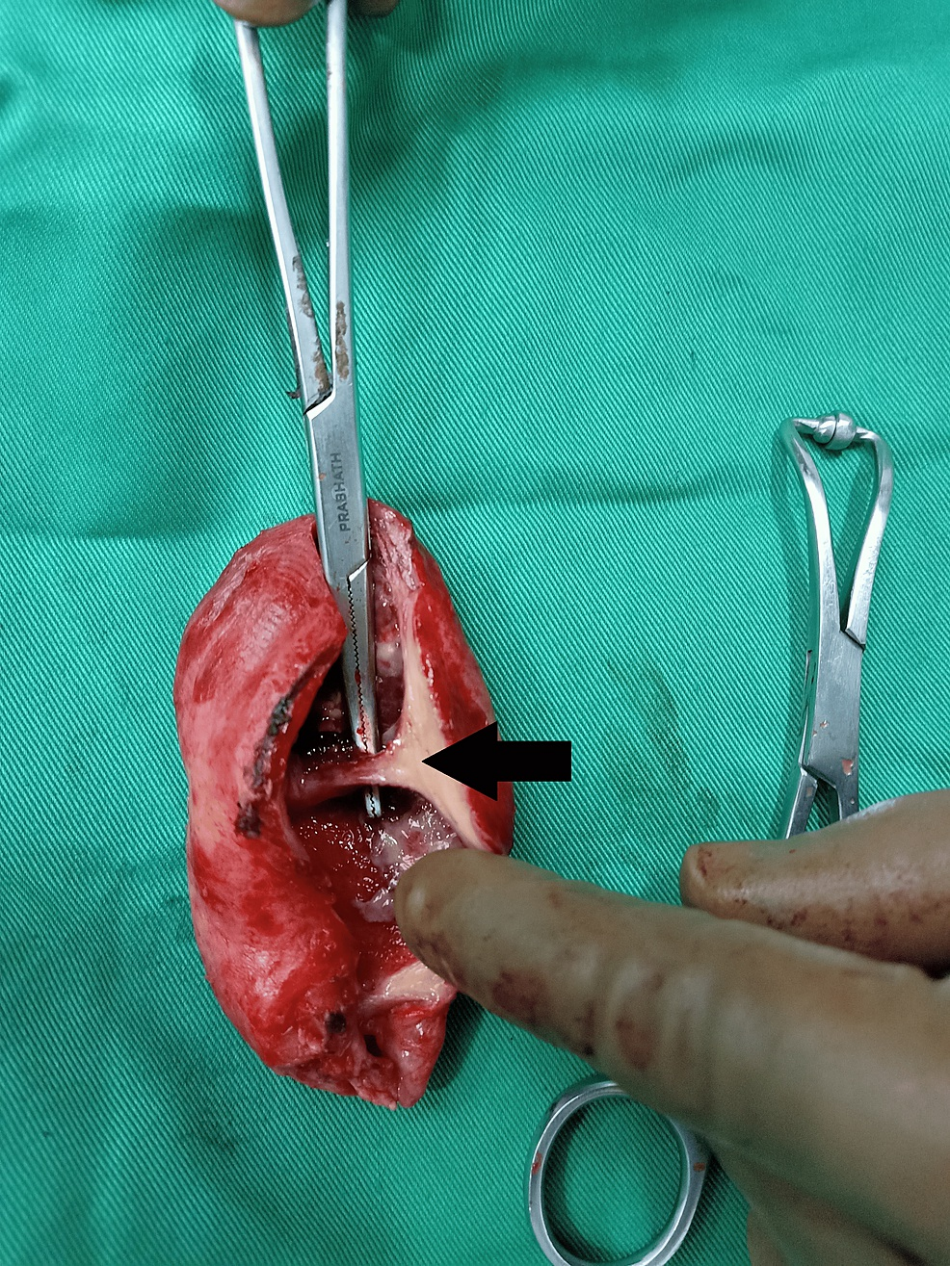
Septate gallbladder Arrow shows the proximal septum separating the lumen of the gallbladder. The mucosa is inflamed, and the wall has grown thicker.

Abdominal ultrasound was recommended, and increased gallbladder thickness in walls was discovered. There was an accumulation of peri cholecystic fluid alongside the edematous wall. The USG, however, did not show septa partitioning the gallbladder lumen into sections. She was diagnosed with a confirmed case of acute cholecystitis and was recommended cholecystectomy. MRCP and any other imaging methods for differential diagnosis of the septate gallbladder were not performed. Following surgery, the gallbladder's macroscopic inspection revealed many edematous septa segmenting its lumen into sections. After surgery, the patient was released without any complications. Following cholecystectomy, she no longer has stomach discomfort or gastrointestinal problems. Figures 1-2 below depict gallbladder septa that were unintentionally discovered after the patient's gallbladder was removed.

## Discussion

A septum that partitions the gallbladder as two halves by a canal is typically cited as a stricture [1], a septate gallbladder [2], and an "hourglass" gallbladder. Less than 150 instances of MSG have been recorded globally since Knetsch first characterized it in 1952 [3]. Unpredictability surrounds the cause of the many septations, although preferred embryologic clarifications involve a firm gallbladder bud's inability to properly vacuolate, chronic wrinkles, or gallbladder bud development that outpaces the bed, causing coiling. It's likewise possible to develop a honeycomb gallbladder, possibly as a result of persistent inflammation. It could be congenital or acquired, without symptoms, and found by chance or identified as an outcome of complaints made by the individual [4]. The solid phase of gallbladder advancement, which exists before the third month of fetal growth, is possibly the cause of the septate gallbladder. Most of the time, gallbladder septae are solitary; however, multiseptate septae, post-inflammatory adhesions, have been additionally seen [5,6]. A theory holds that the numerous septations of the gallbladder are the consequence of septa failing to vanish in the final phases of embryonic growth. The growth begins with the production of an endodermal epithelial bud which would ultimately crease. Then, intraepithelial clefts combine to generate locules, as they broaden, producing septations (walls of locules) surrounding the vacuums they construct. Biopsy observations suggested the existence of smooth muscles in the septations as an extension of the gallbladder wall muscles, which provided evidence in favor of this viewpoint [3]. Different research examined 11 human embryos between 29 days and 25 weeks of pregnancy, and zero had the solid bud or crease occurrence. Rather, they discovered numerous in-pouching of the lumen into the adjacent mucosa roughly in week 12 of pregnancy. It is believed that in the very first trimester, cystic outpouchings represent the beginnings of multi-septations within the gallbladder [7,8].

A Phrygian cap malformation is a congenital septum that is thought to be made up of a mucous membrane fold. Strictures containing muscle that are linked to cholecystitis glandularis proliferans are believed to be acquired [1]. Gallbladder structural variations were classified over the years in multiple manners. Vesica fellea divisa and vesica fellea duplex were identified by Boyden in 1926. The vesica fellea duplex has been further classified into Y- and H-shaped types [6]. Gross categorized dual gallbladder into kinds from A to E in 1936, illustrating the locations of the accessory organ with the dispersion of the cystic ducts [5]. Based on appearance and development, this categorization divides the population into two main groupings (type 1 and type 2). Gallbladders in type 1 cystic primordium divide during growth and have the same cystic duct, while gallbladders in type 2 emerge from distinct primordium and have unique cystic ducts [7].

Cholelithiasis, sludge, cholecystitis, cellular metaplasia, and adenocarcinoma are conditions that are connected to gallbladders. Roeder reported an individual with papillary adenocarcinoma in the second gallbladder and cholecystitis and cholelithiasis in the first [8]. An uncommon genetic abnormality called a septate gallbladder needs careful care. Research has shown that it might be challenging to diagnose the illness radiologically. The physician ought to be conscious of the morphological differences between the gallbladder and biliary system and may discover pre-operative diagnosis problematic. The preferred imaging technique for potential gallbladder anomalies should be MRCP. The dangers of laparoscopic cholecystectomy for septate gallbladders are generally emphasized to be equivalent to those of non-septate gallbladder surgery [9].

Pain in the epigastrium may be unnoticed, and it may be accompanied by cholecystitis or even pancreatitis. Additional bile duct anomalies like choledochal cysts, ectopic gallbladder, or aberrant biliopancreatic junctions could be connected to it. Numerous slender hyperechogenic septa that are at right angles to the walls of the gallbladder and illustrate hypoechogenic cyst-like chambers are seen using ultrasound. Contrast-enhanced ultrasound ought to be regarded as the most reliable method for identifying gallbladder issues [10]. The MSG doesn't need to be taken into consideration as a cause for cholecystectomy since its result is often benign unless it is connected to cholecystopancreatitis and incapacitating chronic discomfort. The disorders on the differential diagnostic list are adenomyomatosis, necrotizing cholecystitis, and hydatid cysts [11].

Gallbladder birth defects are broken down into abnormalities of shape, quantity, place, and size issues. Modern advances in ultrasonography have made it feasible to detect these defects even before birth as soon as the fourteenth week of pregnancy. In a gestational investigation, there were 10,016 fetal gallbladder instances. There were 17 occurrences of fetal abnormalities discovered, including two septate or bilobed gall bladders [12]. Rarely, a septate gallbladder may recur attacks of abdominal pain or cholelithiasis could worsen the condition.

Bile stasis, calculus, and elevated pressure sensations are likely in gallbladders having transverse septa with little connection in the two compartments. With accompanying chronic or acute swelling, septation leads these separate compartments to cholelithiasis. The septate gallbladder is typically unnoticed or observed by accident during the investigation of liver disease or at autopsy examinations, and it can occasionally lead to ultrasound imaging pitfalls and result in a false-positive diagnosis of gallstones [13]. The case of a child with a dual gallbladder and obstructive jaundice was described by Granot et al. [14]. Constant pain in the abdomen from a septate gallbladder might be aggravated by cholelithiasis [15]. An ultrasound of the abdomen revealed that one child in the case report of two children had a septate gallbladder with cholelithiasis, and the other child had frequent episodes of jaundice. An endoscopic retrograde cholangiopancreatography (ERCP) proved the septate gallbladder in both of the children [16,17].

## Conclusions

In conclusion, a peculiar congenital defect called a MSG is an uncommon reason why stomach discomfort recurs. The preferred course of treatment for patients with symptoms, as in our situation, is cholecystectomy. Following surgery, our patients' gastrointestinal issues and stomach discomfort disappeared. Given the potential for undiagnosed gallbladder cancer in older, asymptomatic people, cholecystectomy ought to be taken into consideration.
